# Updates in Management of Unresectable Stage III Non Small Cell Lung Cancer: A Radiation Oncology Perspective

**DOI:** 10.3390/cancers16244233

**Published:** 2024-12-19

**Authors:** Lakshmi Rekha Narra, Ritesh Kumar, Matthew P. Deek, Salma K. Jabbour

**Affiliations:** Department of Radiation Oncology, Rutgers Cancer Institute, New Brunswick, NJ 08901, USA; ln303@rwjms.rutgers.edu (L.R.N.); ritesh.kumar@rutgers.edu (R.K.); deekmp@cinj.rutgers.edu (M.P.D.)

**Keywords:** NSCLC, immunotherapy, EGFR-TKI, chemoradiation

## Abstract

Unresectable stage III non-small-cell lung cancer (NSCLC) remains a clinical challenge, due to the need for optimal local and systemic control. The management of unresectable Stage III NSCLC has evolved with advancements in radiation therapy (RT), systemic therapies, and immunotherapy. For patients with locally advanced NSCLC who are not surgical candidates, concurrent chemoradiotherapy (CRT) has modest survival outcomes, due to both local progression and distant metastasis. Efforts to enhance outcomes have led to dose-escalation trials, advances in modern RT techniques such as intensity-modulated RT (IMRT) and proton beam therapy (PBT), and the integration of adaptive RT to optimize target coverage while sparing organs at risk. Concurrent and consolidative immunotherapy, particularly with PD-L1 inhibitors, has shown promise, as evidenced by the PACIFIC trial, which demonstrated improved progression-free survival (PFS) and overall survival (OS) with durvalumab following CRT. Ongoing trials are now investigating novel immunotherapy combinations and targeted therapies in this setting, including dual checkpoint inhibition, DNA repair inhibitors, and molecularly targeted agents like osimertinib for EGFR-mutated NSCLC. Emerging biomarkers, such as circulating tumor DNA and radiomics, offer potential for personalizing treatment and predicting outcomes. Additionally, PBT and MR-guided adaptive RT have shown the potential to reduce toxicities while maintaining efficacy. Integrating these novel approaches may offer opportunities for optimizing treatment responses and minimizing adverse effects in this challenging patient population. Further investigation into patient stratification, biomarker-driven therapy, and refined therapeutic combinations is essential to improve long-term outcomes in unresectable Stage III NSCLC. This narrative review explores the current management strategies for unresectable Stage III NSCLC, from a radiation oncology perspective.

## Simple Summary

The management of unresectable Stage III non-small-cell lung cancer (NSCLC) has advanced significantly, focusing on enhancing local and systemic control. Current strategies combine radiation therapy (RT), systemic therapies, and immunotherapy. Concurrent chemoradiotherapy (CRT) remains the cornerstone, although outcomes are limited by local progression and distant metastasis. Innovations such as intensity-modulated RT, proton beam therapy, and adaptive RT aim to optimize treatment precision and minimize toxicity. Immunotherapy, particularly PD-L1 inhibitors like durvalumab, has demonstrated improved survival rates, as shown in the PACIFIC trial. Emerging biomarkers, including circulating tumor DNA (ctDNA) and radiomics, offer opportunities for personalized therapy. Research is also exploring novel combinations, such as dual checkpoint inhibitors and targeted therapies, for enhanced effectiveness. Despite challenges in managing toxicities and variability in patient response, integrating these advancements holds promise for improving long-term outcomes in this complex disease.

## 1. Introduction

Lung cancer constitutes 11.7% of all new cancer cases and is estimated to account for 20.4% of all cancer-related mortality in 2023 [1]. Non-small-cell lung cancer (NSCLC), the predominant subtype, comprises 82% of all lung cancer diagnoses, of which approximately 20–30% present with locally advanced disease at the time of diagnosis [2]. For inoperable locally advanced NSCLC, the 5-year historical overall survival remains 20–32%, due to failure from both local disease and distant metastasis, reserving systemic therapy as the mainstay of treatment [3]. Although a universally accepted definition of unresectable disease is lacking, factors to consider include higher clinical tumor stage, lymph node burden, anatomic location, adequate cardiopulmonary reserve, and the technical feasibility of achieving an R0 resection [4]. Moreover, the high risk of distant metastasis and challenges in achieving R0 resection with good functional outcomes often preclude curative surgical approaches in locally advanced T4N0M0 and regionally advanced T3-T4 N1M0 or T1-4 N2-3M0 tumors [5]. Thus, radiation therapy (RT) and systemic therapy play important roles in unresectable Stage III NSCLC for cure, including optimal local control (LC) and distant control. This comprehensive narrative review delves into the current management strategies for unresectable Stage III NSCLC, offering an in-depth exploration of advancements and perspectives from the field of radiation oncology, including innovations in therapy techniques and integrated treatment approaches.

## 2. Radiation in Locally Advanced NSCLC

A clinical trial by Wolf et al. in 1996 suggested a trend toward improved median overall survival (OS) with thoracic RT in locally advanced NSCLC compared to supportive care (4.6 vs. 3.6 months, *p* = 0.17) [6]. The subsequent RTOG 73-01 trial established that continuous fractionation at 60 Gy provided superior local control and fewer intrathoracic recurrences compared to lower doses or split-course regimens [7]. This established the standard tumor control dosing of at least 60 Gy in 30 fractions for locally advanced NSCLC. It is important to note that in these early studies, the predominant histology was squamous cell carcinoma. The limited improvement of 5-year OS by thoracic RT alone in these settings led to the investigation of the role of sequential chemotherapy and RT. The CALGB-8433 trial demonstrated improved median survival with induction chemo followed by thoracic RT, as compared to RT alone (13.7 vs. 9.6 months; *p* = 0.012) [8]. A subsequent phase III trial RTOG 94-10 showed concurrent chemoradiotherapy (cCRT) is superior to sequential chemoradiotherapy (sCRT) approaches (5y-OS: 13–16% vs. 10%, *p* = 0.046) [9]. However, even with concurrent CRT, improvements in survival remained modest, due to local or distant progression of disease, with local progression being 39% in sCRT vs. 30% in cCRT. Similar randomized trials by Furuse et al., Fournell et al., and Zatloukal et al. also showed that cCRT is superior to sCRT [10,11,12]. These findings were further supported by a meta-analysis of six trials which showed an absolute benefit of 5.7% (23.8% vs. 18.1%) in 3 yr OS and 4.5% (15.1% vs. 10.6%) in 5 yr OS (HR, 0.84; 95% CI, 0.74 to 0.95; *p* = 0.004) with cCRT, as compared to sCRT [13].

### Dose Escalation in Locally Advanced NSCLC

Most phase I/II dose escalation trials utilizing 3DCRT investigated high RT doses (65–103 Gy), with improved LC and OS [14,15]. A dose-escalation study by Kong et al. used dose ranges (63–69 Gy, 74–84 Gy, and 92–103 Gy) in patients receiving 3DCRT (without concurrent chemotherapy), with 19% having received neoadjuvant chemotherapy. Improved 5-year OS rates (4%, 22%, and 28%; *p* = 0.0002) and PFS rates (12%, 35%, and 49%) were seen across the dose ranges (63–69 Gy, 74–84 Gy, and 92–103 Gy, respectively) [16]. Similarly, the RTOG 9311 trial with 3DCRT demonstrated that higher doses can be delivered safely in inoperable NSCLC. RT dose was safely escalated to 83.8 Gy in patients with V20 < 25% and 77.4 Gy for those with lung V20 between 25% and 36% [17].

Long-term suboptimal outcomes observed in prior dose-escalation trials using 3DCRT prompted further investigations into the role of dose escalation and more modern techniques such as IMRT in concurrent CRT settings. Recent small studies in the IMRT era have shown that dose escalation can be carried out safely in advanced NSCLC [18,19]. The RTOG 0617 phase III trial (*n* = 544) aimed to assess the benefit of dose escalation (60 Gy vs. 74 Gy) in conjunction with concurrent chemotherapy, also evaluating the addition of cetuximab, an anti-EGFR antibody in a 2 × 2 factorial design [20]. Long-term results showed that dose escalation to 74 Gy did not improve OS, and was associated with decreased median OS (20.3 vs. 28.7 months; *p* = 0.0072), 5-year OS (23% vs. 32.1%; *p* = 0.055), and PFS (13% vs. 18.3%; *p* = 0.055), along with increased toxicity [21]. These findings established concurrent CRT with 60 Gy in 30 fractions as the standard of care for unresectable locally advanced NSCLC with concurrent weekly carboplatin and paclitaxel, followed by two cycles of outback carboplatin and paclitaxel.

## 3. Immunotherapy in Locally Advanced NSCLC

In addition to direct cytotoxicity, CRT alters the tumor microenvironment, facilitating immune cell infiltration and promoting immunogenic cell death. This results in increased tumor antigen presentation and subsequent CD8+ T-cell-mediated killing. However, cancer cells can evade immune surveillance by upregulating immune checkpoint molecules like PD-L1, which binds to PD-1 receptors on T cells, inhibiting their function. Integrating immune checkpoint inhibitors (ICIs) that target PD-L1 disrupts this inhibitory pathway, potentially enhancing the anti-tumor response initiated by RT therapy [22,23]. These agents have led to the changing of the standard of care through the PACIFIC trial, which added consolidative durvalumab for one year after definitive CRT in locally advanced NSCLC compared to placebo [24]. This translated into a substantial increase in 5 yr OS from 33.1% (placebo) to 42.9% with the addition of durvalumab (HR 0.72, 95% CI 0.59–0.89) and 5 yr PFS from 19.0% with placebo to 33.4% with durvalumab (HR 0.55, 95% CI 0.45–0.68), over CRT alone [25]. While well tolerated, the consolidative therapy did lead to a 15.4% discontinuation rate due to pneumonitis and related complications. However, an unplanned subgroup analysis revealed limited benefit in patients with low PD-L1 expression (<1%) and EGFR mutations, highlighting potential areas for further research and personalized treatment approaches; however, these were unplanned subset analyses with limited numbers of patients [26].

To enhance durable responses in unresectable Stage III NSCLC, combination immunotherapy strategies are evolving. RT is known to upregulate immune checkpoints such as CD73, HLA-E (the ligand for NKG2A), and PD-L1 within the tumor microenvironment. Therefore, integrating immune checkpoint inhibitors with chemo-radiotherapy may further enhance antitumor activity. This is supported by preclinical data demonstrating improved efficacy when radiotherapy is combined with anti-CD73 or anti-NKG2A antibodies, alongside PD-L1 inhibition [27,28,29]. Building upon the PACIFIC trial’s success with durvalumab, ongoing research is evaluating novel immunotherapy combinations in consolidation phase to further improve outcomes. The studies of consolidative immunotherapy after CRT are summarized in Table 1.

The COAST trial, a three-arm phase II study (n = 301), evaluated durvalumab plus oleclumab (an anti-CD73 antibody) or durvalumab plus monalizumab (an anti-NKG2A antibody) versus durvalumab alone for unresectable Stage III NSCLC after CRT without progresseion. There were promising PFS rates with the doublet immunotherapy approaches, with 1-year PFS rates of 72.7% (95% CI 58.8–82.6) for durvalumab + monalizumab, 62.6% (95% CI 48.1–74.2) for durvalumab + oleclumab, and 33.9% (95% CI 21.2–47.1) for durvalumab alone [30]. It is noted that the durvalumab alone had lower outcomes than PACIFIC. Similarly, the BTCRC-LUN16-081 trial, a two-arm phase II study (n = 150), evaluated consolidative nivolumab alone versus nivolumab plus ipilimumab in a similar patient population. This study showed improved 18-month PFS (62.3% vs. 67%; *p* < 0.1) and 2-year overall survival (OS) (76.6% vs. 82.8%) with the doublet regimen [31].

Based on these COAST results, the phase III PACIFIC-9 trial is currently underway, investigating durvalumab plus oleclumab, durvalumab plus monalizumab, and durvalumab plus placebo in patients with unresectable Stage III NSCLC who have not progressed after CRT with PFS as primary end point. Other trials, such as PACIFIC-8 and SKYSCRAPER-01, are investigating the addition of anti-TIGIT antibodies to durvalumab, though results have not yet been reported.

Beyond the clinical trial setting, the PACIFIC-R study, a retrospective analysis of 1399 patients across 11 institutions who had early access to durvalumab, demonstrated its efficacy in real-world clinical practice, showing median PFS of 21.7 months, 2 yr PFS of 48.2% and 2 yr OS of 71.2%, including patients with an ECOG PS of 2 and those receiving sCRT [32]. The PACIFIC-6 trial is a phase II study of consolidative durvalumab after sCRT in unresectable Stage III NSCLC. This trial highlighted that sequential CRT (sCRT) followed by consolidative durvalumab is safe, with a grade 3–4 radiation pneumonitis (RP) at 1.7%, which is comparable to 3% observed in the PACIFIC trial [33]. The 1-yr PFS and 1-yr OS were 49.6% and 84.1%, respectively. In addition, a phase III GEMSTONE-301 trial also showed significant improvement in median PFS (9·0 months vs. 5·8 months, *p* = 0·0026) with sugemalimab over placebo, regardless of the chemo-radiotherapy sequence [34].

Patients with poor performance status (PS) are under-represented in clinical trials due to advanced age, comorbidities, and chemotherapy contraindications. Despite these challenges, ongoing studies are evaluating whether immunotherapy combined with RT offers added benefit over RT alone. The SPIRAL-RT trial from Japan, a phase II single-arm study, enrolled patients with PS 2 or those ineligible for chemotherapy, treating them with radiotherapy alone (54–66 Gy) followed by durvalumab consolidation. This trial hypothesized that durvalumab would improve one-year PFS from 16% to 35%. Among the 33 patients enrolled, patients’ median age was 79 years and the study reported a one-year PFS of 39%. Although results are modest, and conducting RCTs in this population remains difficult, the findings indicate potential for safely expanding immunotherapy use in this setting [35].

In summary, combining immunotherapy with CRT has transformed care in unresectable NSCLC, notably with durvalumab consolidation for one year post-CRT with improved survival outcomes, as demonstrated in the PACIFIC trial. Trials like COAST and BTCRC-LUN16-081 show promising PFS results with dual checkpoint inhibition adjuvantly. The ongoing PACIFIC-9 and other trials are exploring novel combinations to enhance efficacy. Real-world data from PACIFIC-R affirm the effectiveness of durvalumab, including in patients with poor performance status and those receiving sCRT. Trials such as SPIRAL-RT also suggest potential benefits for elderly or PS 2 patients, though more research is needed for this subgroup.

### 3.1. Concurrent Immunotherapy with Concurrent Chemoradiation

To enhance treatment outcomes, studies have investigated concurrent administration of immunotherapy with CRT in unresectable Stage III NSCLC. This approach is driven by two key factors: first, a notable percentage (22–30%) of patients experience disease progression or intolerable toxicity during CRT, which precludes them from completing CRT, and are thus ineligible for consolidative durvalumab [9]. Second, preclinical studies indicate that the concurrent use of anti-PD-L1 antibodies with fractionated radiotherapy significantly enhances CD8+ T-cell infiltration within tumors, leading to improved local control, survival, and antitumor immunity when compared to sequential administration [27]. The studies of concurrent immunotherapy with CRT are summarized in Table 2.

Several early-phase studies have examined the concurrent administration of immunotherapy with cCRT. A phase 1 trial by Jabbour et al. evaluated the safety of concurrent pembrolizumab with CRT in 21 patients with Stage III NSCLC. Patients received varying doses of pembrolizumab with after, during, and then on day 1 concurrent CRT. No dose-limiting toxicities occurred across cohorts, though one case of grade 5 pneumonitis arose in the expansion cohort. Grade 3 or higher immune-related adverse events were noted in 18% of patients. Median PFS was 18.7 months, with a 12-month PFS rate of 69.7%. This study indicated that concurrent pembrolizumab with CRT is tolerable with promising PFS, warranting further investigation in larger trials [36]. Nivolumab was also evaluated as concurrent with CRT, followed by consolidative nivolumab in the NICOLAS trial, with a median PFS of 12.7 months [37]. Similarly, the DETERRED trial evaluated atezolizumab with CRT in Stage III NSCLC. Part 1 patients (*n* = 10) received CRT followed by atezolizumab (median PFS 18.9 months, OS 26.5 months), while part 2 patients (*n* = 30) had concurrent and consolidative atezolizumab (PFS 15.1 months, OS not reached). Patients with PD-L1 ≥ 1% had longer PFS (27.4 vs. 11.0 months, HR: 2.01, *p* = 0.10) and OS (not reached vs. 26.5 months, HR: 1.49, *p* = 0.41) than those with PD-L1 < 1%, indicating a trend toward better outcomes with higher PD-L1 expression [38]. These single-arm phase II studies primarily assessed toxicity, particularly grade 3 RP, with rates of 3% in DETERRED and 11.7% in the NICOLAS study suggesting that concurrent immunotherapy with CRT is relatively well-tolerated. KEYNOTE-799 is a phase II study which combined 1 cycle of induction chemotherapy with pembrolizumab, followed by cCRT with pembrolizumab and consolidation with pembrolizumab in unresectable Stage III NSCLC [39]. Cohort A (squamous NSCLC) had a 70.5% objective response rate, and cohort B (non-squamous NSCLC) had a 70.6% response rate, with over 75% of responses lasting 12 months or longer. Grade 3+ pneumonitis occurred in 8.0% of cohort A and 6.9% of cohort B. Treatment-related adverse events of grade 3–5 affected 64.3% and 50.0% in cohorts A and B, respectively. The 1-year PFS was 67.1% in cohort A and 71.6% in cohort B. The study suggests pembrolizumab plus cCRT has strong antitumor activity and manageable safety in locally advanced NSCLC.

Ongoing phase III randomized trials are further investigating the efficacy of concurrent immunotherapy with CRT. The PACIFIC-2 trial compares concurrent durvalumab with CRT followed by durvalumab versus placebo plus CRT followed by placebo in unresectable Stage III NSCLC. Results were recently presented in abstract form, and showed no significant benefit of concurrent durvalumab with CRT. Although not statistically significant, there was a trend toward improved PFS with durvalumab (HR 0.85; *p* = 0.247), with median PFS of 13.8 vs. 9.4 months. Overall survival (OS) showed no significant difference (HR 1.03; *p* = 0.823), with median OS of 36.4 vs. 29.5 months [40]. ECOG-ACRIN 5181 is comparing concurrent durvalumab + cCRT followed by consolidative durvalumab verses cCRT followed by consolidative durvalumab in a phase III study. KEYLINK-012 is exploring the role of concurrent pembrolizumab-CRT followed by consolidative pembrolizumab (with/without olaparib), as compared to CRT followed by consolidative durvalumab. Both ECOG-ACRIN 5181 and KEYLINK-012 have completed accrual and results are awaited. CHECKMATE 73L compared concurrent Nivolumab with cCRT followed by consolidative Nivolumab or Ipilimumab-Nivolumab vs. cCRT followed by consolidative durvalumab. A press release showed that the nivolumab arm did not meet the primary endpoint of PFS. Future studies will continue to explore the addition of other agents, such as anti-TIGIT or olaparib, to immunotherapy, in conjunction with CRT.

### 3.2. Concurrent Immunotherapy with Radiation (No Chemotherapy)

Immunotherapy is being investigated as a potential substitute for chemotherapy when combined with radiation therapy, especially in patients with high PD-L1 expression (Table 3). The rationale for this substitution stems from the superior PFS and OS demonstrated with pembrolizumab in the KEYNOTE studies in patients with high PD-L1 expression [41]. The SPRINT trial investigated a non-chemotherapy treatment regimen in patients with PD-L1 ≥ 50%, utilizing three cycles of induction pembrolizumab, followed by PET-based assessment and risk-adapted hypofractionated RT (48–55 Gy in 20 fractions) without concurrent immunotherapy, and consolidation pembrolizumab for 13 cycles [42]. With a median follow-up of 22 months, this trial had a 1-year PFS of 74%, a median PFS of 26 months, 1-year OS of 92%, and 2-year OS of 76%. Similarly, the DOLPHIN trial investigated the use of concurrent durvalumab and standard RT (60 Gy in 30 fractions), followed by durvalumab consolidation, in patients with any PD-L1 expression [43]. With a median follow-up of 22.8 months, the trial reported a 1-year PFS of 72.1% and a median PFS of 25.6 months. Although the SPRINT and DOLPHIN trials are non-randomized, they demonstrated the feasibility and safety of concurrent immunotherapy with RT in PD-L1-positive patients. However, further phase III randomized controlled trials will be needed to validate these findings.

## 4. Targeted Therapy for Oncogene-Driven Lung Cancer

Modern genetic analysis of NSCLC pathological samples can identify actionable mutations in genes such as EGFR, ALK, and MET, which are targetable with specific therapies. The sub-group analysis of the PACIFIC trial also showed that there is limited benefit of consolidative durvalumab in patients with EGFR mutations [26]. Thus, targeted therapy, such as tyrosine kinase inhibitors (TKIs) can be appropriate in this population. Osimertinib is an oral EGFR-TKI that selectively inhibits both EGFR-TKI-sensitizing and EGFR T790M resistance mutations. It has shown superior efficacy as compared to gefitinib in median PFS (18.9 months vs. 10.2 months; *p* < 0.001) in untreated EGFR-Mutated Advanced NSCLC in the FLAURA study [44]. Similarly, osimertinib demonstrated a survival benefit in adjuvant postoperative setting in resectable NSCLC in the ADAURA trial [45]. The role of consolidative osimertinib after CRT in EGFR mutant Stage III unresectable NSCLC was explored in the phase III LAURA trial [46]. These seminal published results showed significant benefit in median PFS (39.1 months vs. 5.6 months, *p* < 0.001) with indefinite use of osimertinib as compared to placebo. The osimertinib group demonstrated remarkable reductions in new metastases, particularly in the brain (8% with osimertinib vs. 29% without osimertinib) and lung (6% with osimertinib vs. 29% without osimertinib) vs. placebo. Interim OS data showed better 3 year OS with osimertinib (84% vs. 74%, *p* = 0.53) vs. placebo [46]. Nassar et al. performed an international multicenter real-world retrospective analysis of 136 patients with unresectable Stage III EGFR-mutated NSCLC who received CRT followed by treatment with osimertinib, durvalumab, or observation [47]. Osimertinib was superior in the real-world scenario, as the median PFS was not reached with osimertinib while durvalumab and observation had median PFS rates of 12.7 months and 9.7 months, respectively. Concurrent EGFR-TKIs with RT have also been explored in small studies. The WJOG6911L phase II trial from Japan including 27 patients showed that concurrent gefitinib with thoracic RT had a median PFS of 18.6 months and median OS of 61.1 months [48]. Concurrent osimertinib with RT is a potential area of ongoing research with the concern for overlapping toxicities including pneumonitis. NCT05170204 is a phase III multicenter study which will evaluate the efficacy and safety of multiple therapies (alectinib, entrectinib, pralsetinib, and durvalumab) in cohorts of patients with ALK-positive, ROS-1-positive, or RET fusion-positive mutations (respectively) in unresectable Stage III NSCLC. The studies of oncogene-driver targeted drugs in locally advanced NSCLC are summarized in Table 4.

## 5. Combinations with DNA Repair Inhibitors

Poly ADP-ribose polymerase (PARP) inhibitors impair DNA repair mechanisms and enhance RT-induced DNA damage, potentially increasing tumor mutational burden and promoting neoantigen release [49]. Studies have shown that PARP inhibition may upregulate PD-L1 expression in tumor cells [50]. It can activate STING-dependent antitumor immune responses, also [51]. Combining PARP inhibitors with anti-PD-1/PD-L1 agents has demonstrated a favorable safety and efficacy profile across various cancers [52]. In a dose-finding study by Argiris et al., the combination of veliparib with cCRT in NSCLC was feasible and well tolerated [53]. A phase 1 trial involving 48 patients by Kozono et al. evaluated concurrent veliparib with cCRT followed by maintenance veliparib in unresectable Stage III NSCLC, showing good tolerability, with a median PFS of 19.6 months, and a median OS of 32.6 months [54]. However, robust long-term data are lacking on the combination of PARP inhibitors with concurrent RT in NSCLC, as there is a risk of increased radiosensitization and increased normal tissue toxicity. Olaparib is also a PARP inhibitor which is being explored in the KEYLINK-012 study, in which olaparib is being tested in the consolidative phase with/without pembrolizumab.

Additionally, inhibition of NF-κB significantly impairs both homologous recombination (HR) and non-homologous end joining (NHEJ), reducing RT-induced DNA repair biomarkers, thereby enhancing radiosensitivity in NSCLC [55]. Likewise, proteasome inhibitors impede DNA repair and act as radiosensitizers in NSCLC, with synergistic effects [56].

## 6. Modern Radiation Techniques to Improve Outcomes in Unresectable NSCLC

RT is an integral component of management in unresectable Stage III NSCLC. The use of modern techniques such as intensity modulated radiation therapy (IMRT) has been shown to improve the RT dosimetry with a decrease in radiation dose to the lungs and heart. This has been shown to improve the outcomes in NSCLC in the subgroup analysis of the RTOG 0617 study [57]. IMRT can be used for intermediate dose escalation to the GTV in a simultaneous integrated-boost approach. Thus, a lesser cardiac and lung dose can potentially decrease the long-term morbidity and mortality. Similarly, with use of image guidance, the doses to normal lung can be decreased. These advanced tumors shrink during treatment, and, with adaptive RT planning, there is potential to decrease further RT doses to normal organs. In patients with bulky locoregional disease, sequential systemic therapy followed by definitive RT/CRT can be a reasonable approach [8,58]. The modern RT tools of MR-LINAC and proton beam therapy are also being explored to better target the area and decrease the doses to normal organs.

### 6.1. Role of Proton Beam Therapy in Locally Advanced NSCLC

Proton beam therapy (PBT) is being utilized for the treatment of lung cancer (Table 5) [59]. However, it remains one of the most challenging sites to treat with PBT, due to the sensitivity of proton dose distributions to the heterogeneous tissues along the proton path, as well as the impact of respiratory motion. Additionally, there is greater uncertainty when the distal edge of the beam intersects low-density regions of the lung, necessitating adequate treatment margins. Therefore, effective immobilization and motion management techniques, including abdominal compression, breath-hold, and respiratory gating, are crucial considerations [60].

For unresectable Stage III NSCLC, several trials have demonstrated encouraging outcomes with PBT (Table 4). A small phase II study by Oshiro et al. investigated the safety of dose escalation with PBT [61]. In this study, 15 patients with unresectable or medically inoperable Stage IIIA/IIIB NSCLC were treated with 74 Gy RBE (Radio Biological Equivalent) to the primary tumor and 66 Gy RBE to the lymph nodes, with concurrent vinorelbine and cisplatin. Acute ≥ G3 radiation pneumonitis (RP) was observed in two patients, though the authors did not attribute this to PBT, and acute ≥ G3 esophagitis was reported in only one patient. Similarly, a phase II study by Chang et al. included 44 patients with Stage III NSCLC treated with 74 Gy RBE combined with concurrent paclitaxel and carboplatin [62]. In this cohort, acute ≥ G3 RP occurred in one patient (2.27%), and acute ≥ G3 esophagitis was observed in five patients (11.3%). Local recurrence (LR) was reported in nine patients (20.5%), while the 1-year OS and PFS rates were 86% and 63%, respectively.

In a randomized phase II trial by Liao et al., passive-scatter PBT was compared with photon IMRT for treating locally advanced NSCLC [63]. The study found that passive-scatter PBT did not improve dose-volume indices for the lungs, but did result in reduced heart exposure. There was no significant difference in local control rates or the incidence of grade 3 pneumonitis. These findings led to a multi-institutional randomized phase III trial (RTOG 1308; NCT01993810) comparing PBT with photons in inoperable Stage II-IIIB NSCLC. Additionally, a phase II trial is underway to evaluate IMRT with an integrated boost versus PBT with an integrated boost for Stage II-IIIB NSCLC (NCT01629498).

Pencil-beam PBT has been found to reduce radiation doses to normal lung, heart, and other critical organs in the thorax, which is particularly beneficial for patients with large-volume disease, interstitial lung disease, or those requiring re-irradiation [64]. Previous studies, including a subgroup analysis of the RTOG 0617 trial by Chun et al. and another subsequent research study by Atkins et al., have highlighted the impact of cardiac radiation dose on mortality [57,65]. PBT has demonstrated a reduction in cardiac dose, potentially lowering the risk of cardiac morbidity and mortality [63].

Furthermore, effective dose to immune cells (EDIC) was associated with poorer LC and OS in a secondary analysis of the RTOG 0617 trial [66]. EDIC can be calculated using mean heart dose (MHD), mean lung dose (MLD), integral total dose, and the number of treatment fractions. Higher EDIC is linked to worse PFS, severe lymphopenia, and increased risk of radiation pneumonitis (RP) in Stage III lung cancer [67]. PBT has been shown to reduce EDIC compared to photon RT [67].

Carbon-ion radiotherapy (CIRT), which leverages the Bragg peak effect, is gaining attention, and has the potential to influence immune responses. Preclinical studies suggest that CIRT induces complex, hard-to-repair DNA double-strand breaks in irradiated tumor cells [68]. Thus, the combination of immune checkpoint inhibitors (ICIs) with CIRT is a promising area for further investigation.

In summary, PBT offers promising outcomes in lung cancer by reducing doses to critical organs, improving safety, and maintaining efficacy. However, challenges remain, particularly with respiratory motion and dose distribution in lung tissue. Effective motion management is essential. For advanced NSCLC, PBT may lower cardiac and immune-cell doses, potentially enhancing survival. Carbon-ion represents an emerging approach worth further exploration with immunotherapy.

### 6.2. Role of Adaptive RT in Locally Advanced NSCLC

Locally advanced NSCLC demonstrates tumor shrinkage during the course of CRT, with better survival in patients who experience greater reductions in tumor volume during the treatment course [69]. Adaptive radiation therapy (ART) for lung cancer involves modifying the RT plan during the course of RT to accommodate anatomical or physiological changes [70].

In patients with locally advanced NSCLC, CRT with or without immunotherapy, is linked to treatment-related toxicities such as pneumonitis, esophagitis, and long-term cardiac complications. In the PACIFIC trial (CRT followed by consolidative Durvalumab), any grade pneumonitis was seen in 33.9%, with G3-G4 pneumonitis seen in 3.4% [24]. In the NICOLAS study (concurrent nivolumab with definitive CRT), G3-G4 pneumonitis was seen in 11.7% of patients [37]. Similarly, concurrent pembrolizumab with CRT showed 33% ≥ G2 pneumonitis in a phase 1 study [36]. The rate of ≥G3 esophagitis after CRT was 7% on the 60 Gy arm of RTOG 0617, while ≥G2 esophagitis was seen in more than 40% in other studies [20,71]. With ART, the RT dose to the normal OAR can be decreased, which in turn can decrease the potential toxicities of RT including RP, esophagitis, and cardiac toxicities.

ART can be categorized into three types: offline, online, and real-time adaptation [72]. Offline adaptation involves modifying the patient’s treatment plan after administering one or more treatment fractions. This process typically includes re-simulation, re-contouring, and re-planning.

Offline weekly ART for locally advanced NSCLC treated with CRT was prospectively evaluated in the LARITA trial by Ramella et al., involving 50 patients [73]. The study demonstrated reduced toxicity, with acute and late grade ≥3 pneumonitis observed in 2% and 4% of patients, respectively [73]. A recent single-center, small retrospective study on CRT followed by consolidative immunotherapy found that the incidence of any grade RP was similar (20% vs. 19%) in patients with larger tumors receiving ART, compared to those with smaller tumors not receiving ART [74]. Currently, a phase 2 trial (NCT04751747) is underway to prospectively investigate the role of ART in reducing toxicity for patients with locally advanced NSCLC undergoing CRT and immunotherapy.

Online adaptation involves adjusting treatment plans immediately before delivering each fraction, often requiring re-contouring and re-planning based on imaging datasets obtained through IGRT [70]. Adaptive solutions utilizing CBCT, MRI, and PET are currently available. In the case of CBCT-based online ART for NSCLC, stringent dosimetric and quality assurance (QA) protocols are essential, due to variations in tissue density and homogeneity [75].

MR-guided therapy using the MR-LINAC offers superior soft-tissue contrast compared to CT, enabling better assessment of tumor invasion, enhanced target delineation, and real-time tracking of tumor motion [76]. This technology has the potential to aid in ART, improve tumor delineation, tumor dose, and sparing of OAR, and reduce the risk of radiation pneumonitis (RP) and esophagitis. The integration of functional MRI can facilitate ART by providing physiological information and early toxicity biomarkers, enabling more personalized treatment adjustments [77]. Initial studies on MR-guided ART in NSCLC have mainly focused on small tumors treated with SBRT or hypofractionated radiotherapy [78,79]. The PUMA trial, an ongoing multicenter phase 1 study, is currently investigating MRI-guided online ART for locally advanced NSCLC [80].

PET-based ART is an emerging and promising technology. In a phase 2 study, Kong et al. investigated the role of mid-treatment PET-based ART and dose escalation in patients with locally advanced NSCLC receiving concurrent CRT [81]. The study enrolled 42 patients, with a median tumor dose of 83 Gy (ranging from 63 to 86 Gy). The 2-year in-field and overall locoregional tumor control rates were 82% and 62%, respectively, with a 2-year overall survival rate of 52%. The RTOG 1106 trial was planned to validate these findings through a randomized clinical approach [82]. The mean RT dose in the adaptive arm was 71 Gy in 30 fractions. The 2-year local–regional-progression freedom (LRPF) was 59.5% versus 54.6% (*p* = 0.66) for standard versus adaptive RT, while the 3-year OS rates were 49.1% versus 47.5% (*p* = 0.80). The toxicities were similar in both arms. Though there was no benefit of dose-escalated ART, there was no detrimental effect of dose-escalated RT on survival and cardiac events. This approach with consolidative immunotherapy can be explored in future.

Real-time adaptation involves automatically adjusting the treatment plan during a treatment session, using real-time imaging to either gate the treatment beam or track the target with the multi-leaf collimator (MLC) [70]. It has been possible with modern technology, including the use of robotic LINAC or robotic couch or MR-LINAC [83].

## 7. Biomarkers in Unresectable NSCLC

Circulating tumor DNA (ctDNA) is emerging as a critical biomarker in NSCLC, with significant prognostic value. It offers a potential approach for detecting minimal residual disease (MRD) and early recurrence [84]. In a small study by Moding et al., Stage III NSCLC patients with undetectable ctDNA following cCRT demonstrated a 1-year PFS of 80% without consolidative immunotherapy. This PFS rate was not significantly different (*p* = 0.23) from that of patients with undetectable ctDNA who received adjuvant immunotherapy [85]. Additionally, an update from KEYNOTE-799 (Pembrolizumab with CCRT in Stage III NSCLC) revealed that among 46 patients with detectable ctDNA at baseline, 69.6% (32 patients) cleared ctDNA by cycle 7. These patients had improved PFS and OS trends, compared to those with persistent ctDNA [86]

Radiomics has emerged as an important tool in the personalized management of Stage III NSCLC by enabling a deeper understanding of tumor biology through advanced-imaging analysis. This approach extracts quantitative features from medical imaging, such as texture, intensity, and shape, to uncover patterns that are often invisible to the human eye. These radiomic signatures can help predict treatment response, optimize therapeutic strategies, and ultimately improve clinical outcomes. By extracting and analyzing quantitative imaging features, radiomics captures tumor heterogeneity and predicts treatment response. Chetan et al. highlighted radiomics’ potential in identifying predictive biomarkers from imaging, such as kurtosis and texture features, which could forecast treatment outcomes with concurrent chemoradiotherapy [87]. Cook et al. demonstrated that specific textural features derived from pre-treatment 18F-FDG PET/CT scans, including coarseness and busyness, were predictive of response to chemoradiotherapy and correlated with survival, emphasizing radiomics’ prognostic value in stratifying patients [88]. Additionally, Ramella et al. showed that integrating radiomic features with adaptive radiotherapy approaches enhanced prediction accuracy for tumor response during treatment, offering the possibility of real-time therapy modification [89]. Despite these advancements, challenges such as standardization of methodologies and prospective validation is warranted, with comparisons to existing prognostic and predictive markers. However, radiomics holds promise for optimizing therapy and advancing precision medicine in Stage III NSCLC.

## 8. Conclusions

The management of unresectable Stage III NSCLC continues to evolve with the integration of advanced radiation therapy techniques, systemic therapies, and immunotherapy. Recent advancements, such as the use of IMRT, proton beam therapy, and adaptive radiation therapy, have significantly enhanced treatment precision by optimizing target coverage while sparing normal tissues. Immunotherapy, particularly the addition of PD-L1 inhibitors like durvalumab, has redefined the standard of care, as demonstrated by the PACIFIC trial, which showed remarkable improvements in PFS and OS. Similarly, targeted therapies like EGFR-TKIs have demonstrated significant survival benefits in the consolidative setting for patients with EGFR-mutant Stage III NSCLC, as evidenced by the LAURA study. Emerging approaches, including concurrent administration of immunotherapy with chemoradiation and novel combinations such as immunotherapy with DNA repair inhibitors or cCRT with concurrent TKI, hold promise for further improvement in treatment efficacy. These strategies aim to exploit synergistic mechanisms to overcome resistance and improve therapeutic outcomes. Moreover, biomarkers like ctDNA and radiomics are revolutionizing personalized medicine by enabling the prediction of treatment responses, early detection of disease progression, and real-time adaptation of treatment plans based on individual tumor biology. Future research must focus on optimizing therapeutic combinations, integrating precision biomarkers, and exploring innovative strategies like combining systemic therapies. Addressing challenges related to patient selection, access to advanced treatments, and minimizing disparities in care delivery will be critical in achieving equitable and superior outcomes. These efforts will not only enhance survival, but also improve the quality of life for patients with unresectable Stage III NSCLC, transforming the management landscape for this challenging disease.

## Figures and Tables

**Table 1:** Consolidative Immunotherapy Trials in Locally Advanced NSCLC after Radiation Therapy.

Study	Phase	n	Staging	Stage Distribution	Treatment	Primary Endpoints	Results	Factors Affecting Treatment Outcomes			Safety
								Histology	PDL-1	Other Factors	
PACIFIC, NEJM 2017; JCO 2022	III	713	Stage IIIA-IIIB (IASLC Staging Manual in Thoracic Oncology)	IIIA: 52.9% IIIB: 44.7% Other: 2.4%	cCRT [54–66 Gy + platinum-based chemo ≥2 cycles] f/b durvalumab (10 mg/kg IV q 2w) vs. placebo ×1 yr.	PFS, OS	5 yr PFS: 42.9% vs. 33.1% 5 yr OS: 33.4% vs. 19.0%	Sq (HR 0.68, 95% CI: 0.50–0.92) Non-sq (HR 0.45, 95% CI: 0.33–0.59)	25% (HR 0.41, 95% CI: 0.26–0.65) <25% (HR 0.59, 95% CI: 0.43–0.82) Unknown (HR 0.59, 95% CI: 0.42–0.83)	EGFRPositive HR 0.76 (95% CI: 0.35–1.64)Negative HR 0.47 (95% CI: 0.36–0.60)Unknown HR 0.79 (95% CI: 0.52–1.20)	15.4% discontinuation (RP and related toxicities).
PACIFIC-R, JTO 2022	Retrospective	1399	Stage IIIA-IIIB/IIIC (AJCC 7th or 8th)	IA-IIB: 5.3% IIIA: 43.4% IIIB or IIIC: 51.3%	Platinum-based CRT (median RT dose 66 Gy) + consolidative durvalumab (10 mg/kg IV every 2 weeks) for up to 1 year. 52.4%: >60 to ≤66 Gy 41.4%: ≤60 Gy	rwPFS, OS	Median rwPFS: 21.7 mo (95% CI: 19.1–24.5) 2 yr OS: 71.2% (95% CI: 68.8–73.6)	Sq: 14.6 months Non-sq: 25.3 months	≥ 1% (22.4 months) < 1% (15.6 months)	Stage: IIIA (23.7 months) vs. IIIB/IIIC (19.2 months)CRT Type: cCRT (23.7 months) vs. sCRT (19.3 months)Durvalumab Timing: ≤42 days (25.7 months) vs. >42 days (20.8 months)Age: <70 years (22.8 months) vs. 70–75 years (22.4 months) vs. >75 years (19.2 months).	16.5% led to permanent discontinuation. (9.5%-RP/ILD)
PACIFIC-6, JTO 2022	II	117	Stage IIIA-IIIB/IIIC (IASLC staging manual 8th Edition)	IIIA: 37.6% IIIB: 50.4% IIIC: 11.1%	(sCRT): 2+ cycles of platinum-based chemo (cisplatin or carboplatin with vinorelbine, taxane, or pemetrexed) followed by 60 Gy ± 10%) + consolidative durvalumab (1500 mg IV q4weeks). 66.7%: ≥54 to ≤60 Gy and 31.6%: ≥ 60 Gy to ≤ 66 Gy.	G 3-4 PRAE within 6 months	Median PFS: 10.9 mo (95% CI: 7.3–15.6) 1 yr PFS: 49.6% (95% CI: 39.5–58.9) 2 yr OS: 69.8% (95% CI: 5.8–80.2)	NR	1 yr PFS ≥1%: 54.8% <1%: 49.4% 2 yr OS ≥1%:60.2% <1%:70.3%	NR	G 3-4 PRAE within 6 months in 4.3%; 12.8% led to discontinuation due to pneumonitis, 3% discontinuation due to ILD and radiation pneumonitis.
GEMSTONE-301, The Lancet Oncol 2022	III	381	Stage IIIA-IIIB/IIIC(IASLC staging manual 8th edition)	IIIA: 27.8% IIIB: 55.4% IIIC: 16.0% Other: 0.8%	(cCRT or sCRT): 2+ cycles of platinum-based chemo (etoposide, vinorelbine, vinblastine, pemetrexed, taxanes, or gemcitabine; exception, gemcitabine is not allowed in cCRT) with 54–66 Gy RT, followed by sugemalimab 1200 mg IV q3 weeks up to 2 years vs. placebo.	PFS	Median PFS: 9.0 mo (sugemalimab) vs. 5.8 mo (placebo) (HR 0.64, *p* = 0.0026) 1 yr PFS: 45.4% (sugemalimab) vs. 25.6% (placebo).	Sq: 8.31 mo vs. 4.21 mo, HR 0.57 (95% CI 0.41–0.80); Non-Sq: 24.35 mo vs. 9.92 mo, HR 0.77 (95% CI 0.42–1.140).	NR	ChemoRTsCRT 8.08 mo vs. 4.07 mo, HR 0.59 (95% CI 0.39–0.91) cCRT 10.51 mo vs. 6.37 mo, HR 0.66 (95% CI 0.44–0.99)RT Dose:≤60 Gy 10.51 mo vs. 6.21 mo, HR 0.55 (95% CI 0.27–1.12)>60 Gy 8.44 mo vs. 5.39 mo, HR 0.66 (95% CI 0.48–0.90)	Grade 3–4 TRAEs: 9% (sugemalimab) vs. 6% (placebo).Pneumonitis/immune-mediated pneumonitis was most common grade 3–4 AE (3% sugemalimab vs. <1% placebo).
SPIRAL-RT, EJC 2023	II	33	Stage IIIA-IIIB/IIIC	IIIA: 51.5% IIIB: 45.5% IIIC: 3.0%	RT monotherapy (54–66 Gy) in chemotherapy in-eligible patients followed by sequential durvalumab (10 mg/kg IV q2 weeks up to 1 year).	1 yr PFS	Median PFS: 8.9 mo (95% CI: 7.4–19.4 mo) 1 yr PFS: 39.1% (90% CI: 24.7–54.6%, meeting the primary endpoint threshold of 16%).	Sq: 7.4 m Non-Sq: 19.4 m	Median PFS PDL1 < 25%: 7.4 m PDL1 ≥ 25%: 10.2 m Unknown: 19.4 m	EGFR pos: 10.4 mEGFR neg/unknown: 7.7 m	51.5% had radiation pneumonitis, with no cases ≥ Grade 3
LUN14-179, Cancer 2020	II	93	Stage IIIA-IIIB	IIIA: 60% IIIB: 40%	cCRT (cisplatin/etoposide, cisplatin/pemetrexed, or carboplatin/paclitaxel) with 59.4–66.6 Gy followed by consolidative pembrolizumab (200 mg IV Q3W for up to 12 months).	TMDD	Median TMDD: 30.7 mo (95% CI: 18.7 mo to NR); Median PFS: 18.7 mo (95% CI: 12.4–33.8 mo)	NA	NA	NA	Grade ≥ 2 pneumonitis: 10.8%; Grade 3 pneumonitis: 4.3%.
BTCRC LUN 16-081, JCO 2022	II	105	Stage III unresectable		After cCRT: randomized to Arm A: nivolumab (480 mg IV Q4W) for up to 24 weeks or Arm B: nivolumab (3 mg/kg IV Q2W) + ipilimumab (1 mg/kg IV Q6W) for up to 24 weeks.	18-month PFS	Median PFS: 25.8 mo (Arm A), 25.4 mo (Arm B). 18-month PFS: 62.3% (Arm A), 67% (Arm B) (both *p* < 0.1 vs. historical controls)	NA	NA	NA	≥Grade 3 events: 39% (Arm A), 53% (Arm B)
COAST, JCO 2022	II	189	Stage IIIA-IIIB/IIIC unresectable		After cCRT (≥2 cycles platinum chemo [cisplatin or carboplatin] + 54–66 Gy RT) with no progression and randomly assigned 1:1:1. Control Arm: Durvalumab 1500 mg Q4W. Arm A: Durvalumab 1500 mg Q4W + oleclumab 3000 mg Q2W (cycles 1–2), then oleclumab Q4W (cycle 3 onwards). Arm B: Durvalumab 1500 mg Q4W + monalizumab 750 mg q2 wks	ORR	Confirmed ORR: 30.0% (Arm A) vs. 35.5% (Arm B) vs. 17.9% (durvalumab) 1 yr PFS: 62.6% (Arm A) vs. 72.7% (Arm B) vs. 33.9% (durvalumab).	NA	NA	NA	Grade 3–4 TRAEs: 40.7% (Arm A); 27.9% (Arm B); 39.4% (durvalumab); Most common grade 3–4 AEs:Pneumonia (6.8% Arm A, 1.6% Arm B, 9.1% durvalumab); Lymphopenia (6.8% Arm A, 0% Arm B 3% durvalumab).

Abbreviations: DOR, duration of response; EGFR, Epidermal Growth Factor Receptor; ILD, interstitial lung disease; ORR, objective response rate; OS, overall survival; PFS, progression-free survival; rwPFS, real-world progression-free survival; TMDD, time to metastatic disease or death; cCRT, concurrent chemoradiotherapy; sCRT, sequential chemoradiation; CNS, central nervous system; HR, hazard ratio; NA, not available; NE, not evaluable; Non-sq, non-squamous; NR, not reached; PD-1, programmed death-1; PD L-1, programmed death ligand-1; PRAE, possibly related adverse event; Q2W, every 2 weeks; Q3W, every 3 weeks; Q4W, every 4 weeks; RP, radiation pneumonitis; RT, radiotherapy; sCRT, sequential chemoradiotherapy; Sq, squamous; TRAE, treatment-related adverse event; yr, year.

**Table 2:** Immunotherapy with cCRT in locally advanced NSCLC.

Study	Phase	n	Staging	Stage Distribution	Treatment	Primary Endpoints	Results	Factors Affecting Treatment Outcomes			Safety
								Histology	PDL-1	Other Factors	
Jabbour et al. JAMA Oncology, 2020	I	21	Stage III unresectable	IIIA: 38% IIIB: 62%	cCRT (paclitaxel + carboplatin + pembrolizumab + RT 60 Gy/30 fractions) followed by consolidative pembrolizumab	Safety, Toxicity	Median PFS: 18.7 mo 1 yr PFS: 69.7%	NA	No difference in PFS with PDL1 status		Grade ≥3 IRAE–18% Grade 5 pneumonitis–1 patient
NICOLAS, JTO 2020	II	79	Stage IIIA-B unresectable	IIIA: 35.4% IIIB: 63.3% Missing: 1.3%	cCRT (2–3 cycles cisplatin/carboplatin +etoposide/pemetrexed/vinorelbine + RT 66 Gy/33 fractions), followed by nivolumab 360 mg Q3W × 4 (2 cycles with cCRT) followed by nivolumab alone 480 mg Q4W, up to 1 year.	1-yr PFS	Median PFS: 12.7 mo Median OS: 38.8 mo 1-yr PFS: 53.7%	No OS difference between Sq and Non-sq	NA	Stage 2 yr OS: 63.7% (higher for IIIA 81.0% vs. IIIB 55.6%, *p* = 0.037)	Grade ≥3 pneumonitis: 11.7%
DETERRED, Lung Cancer 2022	II	40	Stage III unresectable		Part 1 (Sequential): chemoRT (60–66 Gy with weekly carboplatin and paclitaxel 50 mg/m^2^) followed by consolidation (carboplatin, paclitaxel 200 mg/m^2^, and atezolizumab 1200 mg q3w for 2 cycles) then atezolizumab 1200 mg q3w for up to 1 year if no progression. Part 2 (Concurrent): Atezolizumab 1200 mg q3w concurrent with chemoRT (60–66 Gy with weekly carboplatin and paclitaxel 50 mg/m^2^), followed by the same consolidation as for Part 1.	PFS, OS	Median PFS: 18.9 months (Part 1) vs. 15.1 months (Part 2) HR 1.30 (95% CI: 0.56–3.04, *p* = 0.54) Median OS: 26.5 months (Part 1) vs. NR (Part 2) HR 0.71 (95% CI: 0.25–1.99, *p* = 0.51)	NA	<1%: 11.0 mo >1%: 27.4 mo	Targetable driver oncogene mutation: shorter PFS (9.4 vs. 16.6 months, HR 3.49 (95% CI: 1.19–10.29)	NA
KEYNOTE-799, JAMA Oncol 2022	II	216	Stage III unresectable	IIIA: 37.4% IIIB: 49.1% IIIC: 13.6%	Cohort A (n = 112, sq/non-sq):1 cycle carboplatin/paclitaxel/pembrolizumab, then weekly carboplatin/paclitaxel × 6 cycles + pembrolizumab q3w × 2 cycles concurrent with RT (60 Gy), followed by pembrolizumab 200 mg q3w for up to 1 year. Cohort B (n = 102, non-sq): 3 cycles of cisplatin/pemetrexed + pembrolizumab Q3 wks concurrent with RT (60 Gy) in last 2 cycles, followed by pembrolizumab 200 mg Q3 wks for up to 1 year.	ORR, Grade 3–5 pneumonitis	ORR: 70.5% (cohort A), 70.6% (cohort B) 12-month DOR: 79.7% vs. 75.6% 1-year PFS: 67.1% vs. 71.6% 1-year OS: 81.3% vs. 87%	Cohort A (ORR) Sq: 71.2% Non-sq: 69.2% Cohort B (ORR) Non-sq: 70.6%	Cohort A (ORR) PDL 1 < 1%: 66.7% PDL 1 ≥ 1%: 75.8% Cohort B (ORR) PDL 1 < 1%: 71.4% PDL 1 ≥ 1%: 72.5%	NA	Grade 3–5 pneumonitis: 8% vs. 6.9% Any grade 3–5 TRAE: 64.3% vs. 50%

Abbreviations: DOR, duration of response; EGFR, Epidermal Growth Factor Receptor; ILD, interstitial lung disease; IRAE, immune-related adverse events; ORR, objective response rate; OS, overall survival; PFS, progression-free survival; rwPFS, real-world progression-free survival; TMDD, time to metastatic disease or death; cCRT, concurrent chemoradiotherapy; CNS, central nervous system; HR, hazard ratio; NA, not available; NE, not evaluable; Non-sq, non-squamous; NR, not reached; PD-1, programmed death-1; PD L-1, programmed death ligand-1; PRAE, possibly related adverse event; Q2W, every 2 weeks; Q3W, every 3 weeks; Q4W, every 4 weeks; RP, radiation pneumonitis; RT, radiotherapy; sCRT, sequential chemoradiotherapy; Sq, squamous; TRAE, treatment-related adverse event; yr, year.

**Table 3:** Immunotherapy with RT alone (no chemotherapy) in locally advanced NSCLC.

Study	Phase	n	Staging	Stage Distribution	Treatment	Primary Endpoints	Results	Factors Affecting Treatment Outcomes			Safety
								Histology	PDL-1	Other Factors	
DOLPHIN, JAMA Oncol 2023	II	35	Stage IIIA-IIIB/IIIC(IASLC staging manual 8th)	Post-operative recurrence: 9% IIIA: 45.7% IIIB: 20% IIIC: 8.6%	Concurrent durvalumab (10 mg/kg IV q2weeks) with 60 Gy radiation therapy 3D-CRT: 70% IMRT: 11%	1 yr-PFS	Median PFS: 25.6 mo (95% CI: 13.1 mo to NE) 1 yr PFS: 72.1% (90% CI: 59.1–85.1%)	NA	NA	NA	Grade 3–4 AEs in 52.9%, including pneumonitis, radiation pneumonitis in 11.8%.
SPRINT, JCO 2023	II	25	Stage IIIA-IIIB/C	II:14% IIIA: 52% IIIB/IIIC: 44%	PD-L1 (TPS) score >50%: Induction pembrolizumab (200 mg IV q3W × 3 cycles) + risk-adapted radiotherapy (MTV < 20cc: 48 Gy and 55 Gy for larger lesions) in 4 weeks, then maintenance of pembrolizumab up to 1 yr <50%: Standard chemoRT followed by adjuvant durvalumab or osimertinib	1-year PFS rate	Median PFS: 26 months 1 year PFS: 76% 2 year OS: 76%	NA	all had PDL1 ≥ 50%	Partial/complete response rate after induction pembrolizumab: 48%; Median MTV Induction immunotherapy:36cc Standard chemoRT: 26cc	Grade 3 AEs: colitis (8%), esophagitis (4%) No grade 4–5 treatment-related AEs

Abbreviations: DOR, duration of response; EGFR, Epidermal Growth Factor Receptor; ILD, interstitial lung disease; IRAE, immune-related adverse events; ORR, objective response rate; OS, overall survival; PFS, progression-free survival; rwPFS, real-world progression-free survival; TMDD, time to metastatic disease or death; cCRT, concurrent chemoradiotherapy; CNS, central nervous system; HR, hazard ratio; MTV, metabolic tumor volume; NA, not available; NE, not evaluable; Non-sq, non-squamous; NR, not reached; PD-1, programmed death-1; PD L-1, programmed death ligand-1; PRAE, possibly related adverse events; Q2W, every 2 weeks; Q3W, every 3 weeks; Q4W, every 4 weeks; RP, radiation pneumonitis; RT, radiotherapy; sCRT, sequential chemoradiotherapy; Sq, squamous; TRAE, treatment-related adverse event; yr, year.

**Table 4:** Studies with oncogenic-driver mutations in locally advanced NSCLC.

Study	Phase	n	Staging	Stage Distribution	Treatment	Primary Endpoints	Results	Safety
WJOG 6911L, JTO 2021	II	27	Stage III unresectable with EGFR mutation	IIIA: 52% IIIB: 48%	Gefitinib 250 mg daily + Thoracic RT (64 Gy/32 fractions) followed by Gefitinib maintenance up to 2 years.	PFS	Median PFS: 18.6 months (95% CI, 12–24.5) Median OS: 61.1 months 2-year PFS rate: 29.6%	Pneumonitis Grade 1–2: 89% Grade 3–4: None Liver dysfunction Grade 3: 44% Grade 4: 15%
LAURA, NEJM 2024	III	216	Stage III unresectable with EGFR mutation	IIIA: 35.2% IIIB: 48.6% IIIC: 16.2%	cCRT followed by Osimertinib vs. Placebo	PFS	Median PFS: 39.1 m vs. 5.6 m (*p* < 0.001) 1 yr PFS—74% vs. 22%	Grade3 tox: 35% vs. 12% RP (any)–48% vs. 38%
Nassar et al, JTO, 2024	Retrospective	136	Stage III unresectable with EGFR mutation	IIIA: 38.2% IIIB: 50.0% IIIC: 11.8%	cCRT followed by Osimertinib vs. Durvalumab vs. Placebo	PFS	Median PFS: Osimertinib—NR Durvalumab—13.7 m Placebo—9.7 m (*p* < 0.0001)	Grade3 Tox: Osimertinib–6.1% Durvalumab–18%

Abbreviations: cCRT, concurrent chemoradiotherapy; DOR, duration of response; EGFR, Epidermal Growth Factor Receptor; ILD, interstitial lung disease; ORR, objective response rate; OS, overall survival; PFS, progression-free survival; HR, hazard ratio; NA, not available; NE, not evaluable; NR, not reached; Q2W, every 2 weeks; Q3W, every 3 weeks; Q4W, every 4 weeks; RP, radiation pneumonitis; RT, radiotherapy; sCRT, sequential chemoradiotherapy; yr, year.

**Table 5:** Studies with proton beam therapy in locally advanced NSCLC.

Study	Phase	n	Staging	Treatment	Primary Endpoints	Results	Safety
Chi et al., Radiotherapy & Oncology 2017	Meta-analysis	72 SBRT studies (7291 patients), 9 PBT studies (614 patients)	Stage I NSCLC (T1, T2, or T3, N0, M0) as per AJCC 7th	PBT vs. SBRT	OS PFS LC Toxicity	3-year LC (87.4% vs. 86.1%, *p* = NS) 3-year OS (69.5% vs. 58.8%, *p* < 0.05)	G3 RP PBT 0.9% vs. SBRT 3.4%; *p* < 0.001 Rib fractures PBT 13% vs. SBRT 3.2%; *p* < 0.001
Oshiro et al., Journal of Radiation Research 2014	Phase II	15	Stage III NSCLC IIIA: 27% IIIB: 73%	PBT (74 Gy RBE to primary tumor, 66 Gy RBE to lymph nodes; adaptive planning; concurrent cisplatin and vinorelbine)	Toxicity OS PFS LC RR	2 yr OS: 51%. Median PFS: 10.2 months Local recurrence in 6 patients (40%)	≥G3 RP—2 patients ≥G3 esophagitis—1 patient
Chang et., Cancer 2011	Phase II	44	Stage III NSCLC as per AJCC staging IIIA: 47.7% IIIB: 52.3%	PBT (74 Gy RBE) with weekly carboplatin and paclitaxel (50 mg/m^2^)	Median OS	Median survival: 29.4 months 1-year OS: 86% 1-year PFS: 63% Local recurrence: 20.5%	G3 dermatitis (11.4%) G3 esophagitis (11.4%) G3 pneumonitis (2.3%)
Liao et al., JCO 2018	Phase II Randomized	149 (IMRT: 92, PSPT: 57)	Stage IIB-IIIB NSCLC IIA/B: 9.4% IIIA: 43.6% IIIB: 34.9% IV: 5.4% Recurrence: 6.7%	PSPT or IMRT concurrent platinum and taxane-based chemotherapy	Grade ≥ 3 RP Local failure	Local failure: IMRT 10.9% vs. PSPT 10.5%. However, there was a significant reduction in RT-dose exposure to the heart.	Grade ≥ 3 pneumonitis: IMRT 6.5% vs. PSPT 10.5%.

DOR, duration of response; ILD, interstitial lung disease; IMRT, intensity-modulated radiation therapy; LC, local control; ORR, objective response rate; OS, overall survival; PFS, progression-free survival; cCRT, concurrent chemoradiotherapy; HR, hazard ratio; NA, not available; NE, not evaluable; NR, not reached; PBT, proton beam therapy, PSPT, passive-scattering proton therapy; Q2W, every 2 weeks; Q3W, every 3 weeks; Q4W, every 4 weeks; RP, radiation pneumonitis; RR, recurrence rate; RT, radiotherapy; SBRT, stereotactic body radiation therapy; sCRT, sequential chemoradiotherapy; yr, year.

## Data Availability

This is a review article with all data taken from web-based resources. No research data exist for this study.
